# Antibody-Mediated Delivery of VEGF-C Promotes Long-Lasting Lymphatic Expansion That Reduces Recurrent Inflammation

**DOI:** 10.3390/cells12010172

**Published:** 2022-12-31

**Authors:** Nikola Cousin, Sophie Bartel, Jeannette Scholl, Carlotta Tacconi, Annina Egger, Gudrun Thorhallsdottir, Dario Neri, Lothar C. Dieterich, Michael Detmar

**Affiliations:** 1 Institute of Pharmaceutical Sciences, Swiss Federal Institute of Technology (ETH) Zurich, 8093 Zurich, Switzerland; 2 Department of Biosciences, University of Milan, 20133 Milan, Italy; 3 Philochem AG, 8112 Otelfingen, Switzerland; 4 European Center for Angioscience (ECAS), Medical Faculty Mannheim, Heidelberg University, 68167 Mannheim, Germany

**Keywords:** lymphatic vessel, VEGF-C, targeted delivery, dermatitis, psoriasis, recurrence

## Abstract

The lymphatic vascular system plays a fundamental role in inflammation by draining interstitial fluid, immune cells, antigens, and inflammatory mediators from peripheral tissues. Site-specific delivery of the lymphangiogenic growth factor VEGF-C alleviates acute inflammation in mouse models of psoriasis and chronic colitis by enhancing local drainage. However, it is unclear whether therapeutically induced lymphangiogenesis is transient or long-lasting and whether it might prevent relapses of inflammation. Here, we investigated the long-term effects of targeted VEGF-C delivery in a chronic dermatitis model in mice. Congruent with our previous results, intravenous injection with a VEGF-C fusion protein targeted to the EDA domain of fibronectin initially resulted in reduced inflammation. Importantly, we found that targeted VEGF-C-mediated expansion of lymphatic vessels in the skin persisted for more than 170 days, long after primary inflammation had resolved. Furthermore, the treatment markedly decreased tissue swelling upon inflammatory re-challenge at the same site. Simultaneously, infiltration of leukocytes, including CD4+ T cells, macrophages, and dendritic cells, was significantly reduced in the previously treated group. In conclusion, our data show that targeted delivery of VEGF-C leads to long-lasting lymphatic expansion and long-term protection against repeated inflammatory challenge, suggesting that it is a promising new approach for the treatment of chronic, recurrent inflammatory diseases.

## 1. Introduction

The lymphatic vasculature plays an essential role in tissue homeostasis and immune surveillance. In inflammatory conditions, tissue remodeling and expansion of blood vessels (angiogenesis) facilitate immune cell infiltration into the affected tissue and fluid extravasation, resulting in tissue edema. On the other hand, lymphatic vessels (LVs) serve as unidirectional drainage routes for accumulating interstitial fluid, thereby reducing edema. In addition, LVs facilitate the transport of immune cells, inflammatory mediators, and antigens to draining lymph nodes (LNs), where adaptive immune responses against tissue-derived antigens may be triggered [1]. While acute inflammation is often required for an adequate host response to, e.g., injury or infection and quickly resolves afterwards, chronic inflammatory diseases, such as psoriasis and atopic dermatitis, are characterized by non-resolving and/or recurring inflammatory episodes, often at the very same tissue site [2,3].

Vascular endothelial growth factors A and C (VEGF-A and -C) are among the most important mediators of the expansion of blood and lymphatic vessels. Notably, these two factors have contrary effects on the course of inflammation. While enhanced VEGF-A expression aggravated inflammatory conditions due to increased edema [4,5], VEGF-C has been shown to ameliorate the course of inflammation in several chronic disease models in mice, such as chronic dermatitis [6], chronic colitis [7], and rheumatoid arthritis [8]. VEGF-C mainly signals via VEGFR-3, a receptor that is predominantly expressed by lymphatic endothelial cells (LECs). Activation of VEGFR-3 reduces skin inflammation, epidermal hyperplasia, and immune cell infiltration by enhancing lymphatic drainage capacity. In contrast, inhibition of VEGFR-3 leads to aggravation of these symptoms in several mouse dermatitis models [9]. Interestingly, VEGF-C may not only act by stimulating lymphangiogenesis, but may also strengthen barrier functions in LECs in vitro [10], which might contribute to the enhanced drainage capacity of LVs that may become leaky and dysfunctional in chronic inflammatory skin conditions [11]. Therefore, activation of the lymphatic vasculature via the VEGF-C/VEGFR-3 axis represents a promising therapeutic approach for the treatment of chronic inflammation.

Previous approaches to therapeutic VEGF-C application have been limited by the need for viral gene delivery systems and/or a lack of targeting strategies [12]. We were able to circumvent these challenges by developing a fully humanized VEGF-C antibody fusion protein (F8-VEGF-C), which delivers VEGF-C to sites of inflammation by binding to the extra domain A (EDA) of fibronectin (FN). EDA fibronectin expression is confined to tissues undergoing active remodeling, such as inflamed skin. Indeed, EDA expression was found in psoriatic skin lesions, making it a highly specific target [6,7,13]. In two psoriasis-like skin inflammation models, an oxazolone-mediated contact hypersensitivity (CHS) model in mice overexpressing VEGF-A in the epidermis [14] and the repeated application of imiquimod in wild-type mice [15], we demonstrated that systemic injection of F8-VEGF-C potently reduced edema and inflammation by spatially restricted lymphangiogenesis induction and enhanced lymphatic drainage compared to the F8-SIP control injected mice, in which the protein targets the same moiety but is inert [6]. However, the longevity and the long-term impact of therapeutically induced lymphangiogenesis on recurrent inflammation at the same site have not been studied yet.

Here, we investigated the long-term effects of F8-VEGF-C application using an oxazolone-driven CHS mouse model of recurrent inflammation. Treatment with the F8-VEGF-C fusion protein resulted in a stably expanded lymphatic network in the affected skin and long-term anti-inflammatory activity that persisted for more than 70 days, reducing the severity of edema and immune cell infiltration upon hapten re-challenge. Thus, targeted, antibody-mediated delivery of VEGF-C is a promising therapeutic approach for the clinical management of chronic, recurrent inflammatory diseases.

## 2. Materials and Methods

### 2.1. Expression of Fusion Proteins

Stably transfected FreeStyle CHO-S cells (R80007, Thermo Fisher, Waltham, MA, USA) expressing the F8-VEGF-C fusion protein were cultured in PowerCHO-CD2 medium (BELN12-771Q, Lonza, Basel, Switzerland) supplemented with 8 mM L-glutamine (25030081, Gibco, Thermo Fisher, Waltham, MA, USA) and 1× ProHT supplement (BE17-855E, Lonza, Basel, Switzerland). Cells were incubated at 37 °C. For production, cells with a density of 4 × 10^6^ were transferred into ProCHO4 (BEBP12-029Q, Lonza) supplemented with 8 mM L-glutamine and 1× ProHT supplement. Cells were incubated at 31–32 °C over a period of 5–7 days.

As for the F8-SIP, FreeStyle CHO-S cells (Thermo Fisher) were cultured at 37 °C to a concentration of 4 × 10^6^ cells/mL in PowerCHO-2CD medium (Lonza) and transiently transfected using PEI (polyethylenimine) in Pro-CHO-4 (Lonza) with plasmids (0.8–0.9 µg/10^6^ cells) encoding the F8-SIP. For production, cells with a density of 4 × 10^6^ were transferred into ProCHO4 (BEBP12-029Q, Lonza) supplemented with 8 mM L-glutamine and 1X ProHT supplement. Cells were incubated at 31–32 °C over a period of 5–7 days.

### 2.2. Protein Purification

CHO-S supernatant was filtered (0.22 μm pore size) and subjected to affinity chromatography using protein A (29-0588-05, Antibody package Protein A, Cytiva, Marlborough, MA, USA). Elution was achieved at 0.1 M glycine pH3. The eluted fraction was then purified with a Spectra/Por membrane tube (128058, Thermo Fisher) in sterile PBS for 2–3 days. Afterwards, the protein eluate was filtered again (0.22 μm pore size), fractioned, and subsequently shock frozen.

### 2.3. Immunofluorescence

Ears of K14-VEGF-A transgenic mice were harvested, embedded in OCT, and snap-frozen on liquid nitrogen. Ears were then sectioned at 7 μm and prepared for immunofluorescence. Sections were fixed in acetone (−20 °C) for 2 min and rehydrated in 80% methanol (4 °C) for 5 min, followed by blocking (5% donkey serum, 1% bovine serum albumin, 0.05% Triton-X, and 0.05% NaN_3_ in PBS). Sections were incubated with primary antibodies in blocking solution over night at 4 °C. The following antibodies were used: anti-LYVE-1 (11-032, AngioBio, San Diego, CA, USA, 1:600), anti-CD31 (550274, BD Biosciences, San Diego, CA, USA, 1:200), anti-CD45 (AF114, R&D Systems, Minneapolis, MN, USA, 1:100), anti-CD68 (ab53444, Abcam, Cambridge, UK, 1:200), anti-CD4 (4SM95, Thermo Fisher, 1:100), and anti-CD206 (AF2535, R&D, 1:20), anti-F4/80 (ab6640, Abcam, 1:100). After washing, secondary antibodies: donkey anti-rat Alexa 488 (A-21208, Thermo Fisher, 1:200), donkey anti-goat Alexa 594 (A-11058, Thermo Fisher, 1:200), donkey anti-rat Alexa 594 (A-21209, Thermo Fisher, 1:200), donkey anti-rabbit Alexa 488 (A-21206, Thermo Fisher, 1:200), and Hoechst 33342 (H3570, Thermo Fisher, 1:5000) were applied in PBS for 20–30 min at room temperature, followed by mounting with Mowiol mounting medium.

### 2.4. Image Analysis

At least 3–5 images were acquired per animal with an Axioskop2 mot plus microscope (Carl Zeiss, Oberkochen, Germany) with an AxioCam MRc camera (Carl Zeiss) and a Plan-APOCHROMAT ×10 or ×20 objective (Carl Zeiss). Regions of interest were defined as the area between the basal membrane of the epidermis and the central cartilage. Fiji was used for image quantification [16]. Results are displayed as either positive area or number of vessels normalized to 1 mm of the basement membrane length. 

### 2.5. Mouse Models

All animal experiments were performed in accordance with animal protocols (ZH212/19) approved by the local veterinary authorities (Kantonales Veterinäramt Zürich, Zurich, Switzerland). Female hemizygous K14-VEGF-A transgenic mice were bred at the ETH Zurich rodent facility under specific pathogen-free conditions. At least 5 mice per group aged between 8–10 weeks were subjected to topical sensitization with 2% oxazolone (Sigma-Aldrich, St. Louis, MI, USA) in 4:1 acetone: olive oil on their shaved abdomen (50 μL) and on each paw (5 μL). On the 5th day after sensitization, mice were topically challenged with 1% oxazolone (Sigma-Aldrich) in 4:1 acetone: olive oil on both sides of both ears (10 μL). Ear thickness was periodically monitored with a caliper. Starting on day 7 post-challenge, mice were treated intravenously with either F8-VEGF-C or F8-SIP (50 μg resp. 2.5 mg/kg body weight) every other day until day 13 (four treatments in total). Mice were euthanized after the inflammation had resolved. 

For hapten re-challenge, mice were again treated topically with 1% oxazolone (Sigma-Aldrich) in 4:1 acetone: olive oil on both sides of both ears (10 μL) once primary inflammation had been resolved. On day 10 after the recall challenge, mice were euthanized.

### 2.6. Flow Cytometry

On the day of euthanization, one ear per mouse was harvested, followed by mechanical disruption. Tissue was digested under rotation in DMEM (41965-039, Gibco) at 37 °C with 4 mg/mL collagenase IV (17104019, Gibco). Tissue was filtered using 40 μm strainers and resuspended in FACS buffer (1% FBS, 1 mmol/L EDTA, and 0.02% NaN_3_ in PBS) to achieve a single-cell suspension. Samples were treated with anti-CD16/CD32 (101302, BioLegend, San Diego, California, USA, 1:100) for 20 min on ice followed by antibody staining. To analyze immune cell populations, cells were stained with anti CD45-PacificBlue (103216, Biolegend, 1:400), anti CD4-APC (100412, Biolegend, 1:400), anti γδ-TCR-PerCp AF700 (100714, eBioscience, San Diego, CA, USA, 1:400), anti CD8-APC/Cy7 (46-5711-82, Biolegend, 1:400), anti CD11b-BV605 (101257, Biolegend, 1:200), anti F4/80-PE (12-4801-82, eBioscience, 1:200), anti Ly6G-FITC (551460, BD Biosciences, 1:400), anti MHCII-AF700 (107622, Biolegend, 1:800), anti CD11c-PE/Cy7 (117318, Biolegend, 1:400), anti Foxp3-PE/eFlour610 (61-5773-82, Thermo Fisher, 1:200) and Zombie-Aqua (423102, Biolegend, 1:800) on ice for 20 min. Data were acquired on a CytoFLEX S flow cytometer (B96621, Beckman Coulter, Brea, CA, USA) using FACS Diva 6.1.3 and analyzed using FlowJo v10.5.3 (BD Biosciences).

### 2.7. Clearance Assay 

To assess lymphatic clearance capacity, we used the polyethylene glycol (PEG)–coupled lymphatic-specific tracer P20D800 (PEG amine P20 conjugated to IRDye800 (929-70020, LI-COR Biosciences, Lincoln, New England, USA)) [17]. Mice were anesthetized with isoflurane (2%), and 3 μL of a 3 μM tracer was injected intradermally into the ear skin, and mice were placed into the IVIS Spectrum in vivo imaging system for image acquisition. Image settings were set to a 2 s exposure time (λ_ex_: 745 nm, λ_em_: 800 nm, binning of 4). Upon tracer injection, mice were imaged immediately (timepoint 0) and then at the timepoints 1 h, 2 h, 3 h, 4 h, and 6 h. Mice were anesthetized to a minimum to prevent isoflurane-dependent inhibition of lymphatic pumping. An additional background image was acquired to adjust the intensity. Analysis was performed by normalizing all timepoints to timepoint 0 and using a 1-phase exponential decay model [18]. The decay constant *K* (expressed in h^−1^) and half-life (expressed in h) were computed using the equation “normalized tissue intensity = *e*^−Kt^”, where *e* is Euler’s number and *t* is time.

### 2.8. Statistical Analysis

All statistical analyses were performed in Prism (9.3.0, GraphPad Software, LLC, San Diego, CA, USA). Results are described as mean ± standard deviation (SD), and statistical significance was determined either by an unpaired Student’s t test (comparing two groups) or with a 2-way ANOVA (comparing two or more groups with repeated measurements in a time dependent matter) with Bonferroni corrected multiple comparison (for the visualization of significant differences in individual measure timepoints between the groups). 

## 3. Results

### 3.1. Targeted Delivery of VEGF-C Potently Reduces Ear Skin Edema and Induces Persisting Lymphatic Vessels in CHS-Induced Skin Inflammation

**Figure 1:**
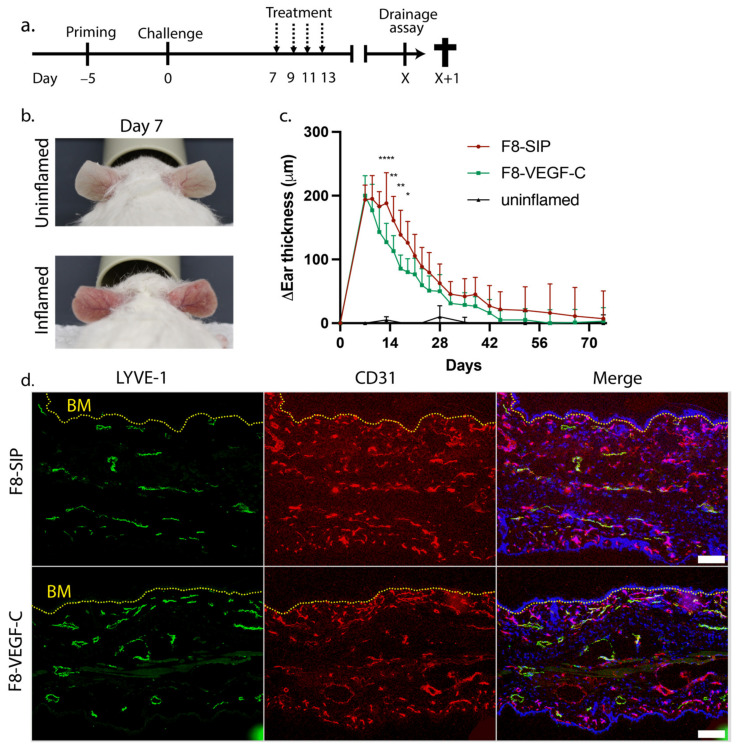

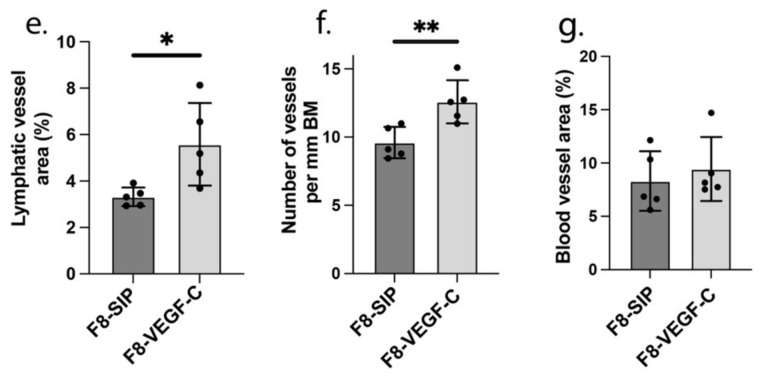
(**a**) Schematic representation of the experimental setup with the treatment regimen on days 7, 9, 11, and 13 (dashed arrows). X indicates the day when ear thickness in both treatment groups returned to baseline, and therefore the day when inflammation was resolved. (**b**) Representative images of uninflamed versus inflamed ears in K14-VEGF-A transgenic mice 7 days after CHS-induced inflammation. (**c**) The ear thickness of F8-SIP and F8-VEGF-C-treated mice compared to pre-challenge thickness (*n* = 10 mice per group, 2-way ANOVA with Bonferroni post-test) until day 74. Ear thickness of uninflamed mice is shown for comparison (*n* = 3). (**d**) Representative immunofluorescence images of ears from F8-SIP and F8-VEGF-C-treated mice stained for the lymphatic marker Lyve-1 (green), the vessel marker CD31 (red), and Hoechst 33342 (blue) on day 74 post-challenge. The dotted yellow line indicates the basement membrane (BM). Size bars: 100 μm. (**e**) Quantification of lymphatic vessel area in % of the analyzed region of interest in F8-SIP and F8-VEGF-C-treated mice on day 74 post-challenge (expressed as mean %, *n* = 5 mice per group, two-tailed Student’s *t*-test). (**f**) Analysis of the number of lymphatic vessels in F8-SIP and F8-VEGF-C-treated mice in the region of interest on day 74 post-challenge (expressed as mean number per mm basement membrane length, *n* = 5 mice per group, two-tailed Student’s *t*-test). (**g**) Quantification of the blood vessel area as a % of the analyzed area on day 74 post-challenge (expressed as mean %, *n* = 5 mice per group, two-tailed Student’s *t*-test). One representative of two independent experiments is shown. All data are presented as mean ± SD. * *p* < 0.05, ** *p* < 0.01, **** *p* < 0.0001.

We induced inflammation in the ear skin of hemizygous K14-VEGF-A transgenic mice using a hapten (oxazolone)-driven CHS model [6,19]. Then, we treated these mice intravenously every second day starting on day 7 until day 15 post-challenge with either a non-functional control protein (F8-SIP) [13] or the therapeutic protein (F8-VEGF-C) delivering VEGF-C to the EDA-domain of fibronectin [6]. We followed the course of the inflammatory response by measuring ear thickness until resolution (Figure 1a–c). Targeted VEGF-C delivery significantly reduced ear skin edema by day 13 post-challenge (day 6 after the start of the treatment) compared to the control protein. Ear edema resolved completely in F8-VEGF-C-treated mice by day 59 post-challenge, while it remained elevated for a longer time period and resolved only by day 74 in F8-SIP control-treated mice (Figure 1c). Previously, we have shown that F8-VEGF-C induces local expansion of the lymphatic vasculature by day 8 after the start of the treatment [6]. To investigate whether this expansion persisted even after inflammation had resolved, we performed immunofluorescence staining of ear sections and analyzed both lymphatic and blood vessels in the ears on day 74 (Figure 1d) as well as on day 179 (Supplementary Figure S1a). Interestingly, we still observed an increased lymphatic vessel area and number on day 74 as well as on day 179 in mice that were treated with F8-VEGF-C even at these late timepoints (Figure 1e,f, Supplementary Figure S1b,c), whereas the blood vessel area at day 74 remained unchanged (Figure 1g).

### 3.2. F8-VEGF-C Treatment Does Not Significantly Affect Lymphatic Drainage Nor Immune Cell Distribution 74 Days Post-Challenge

**Figure 2:**
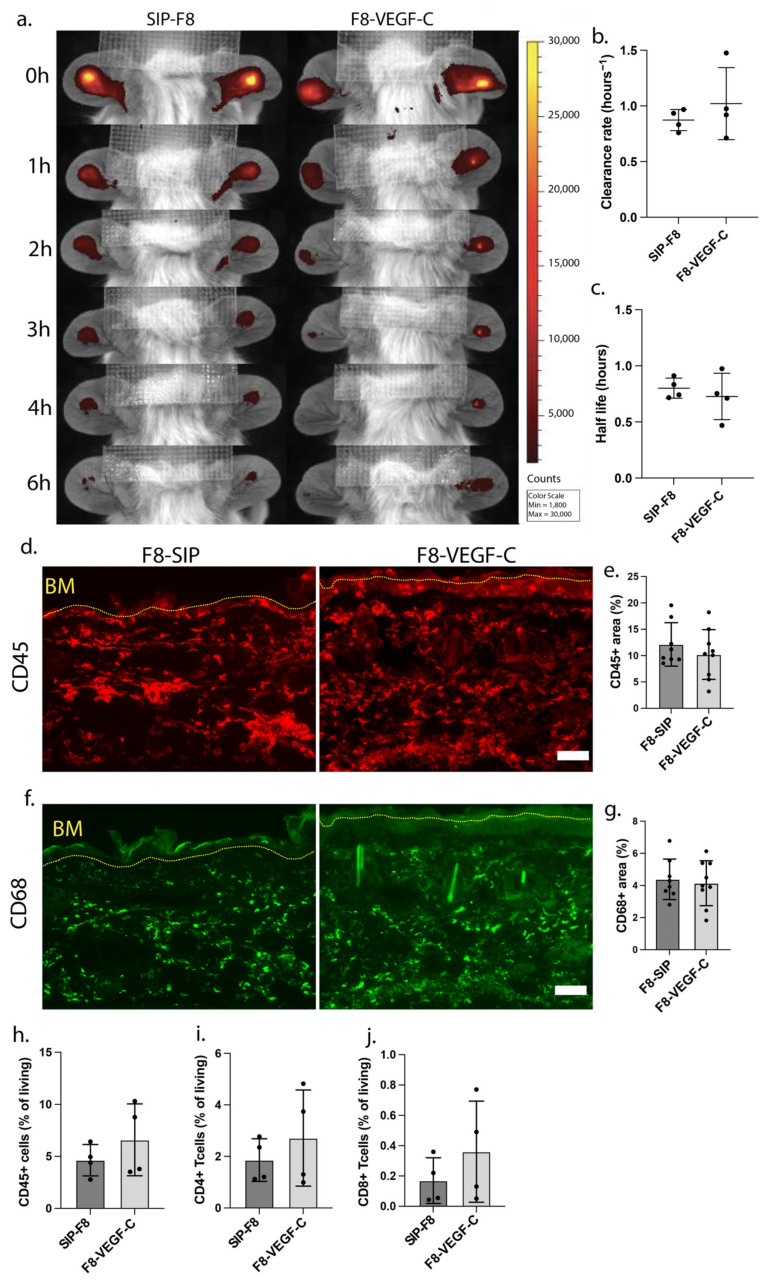
(**a**) Representative images taken by the in vivo imaging system (IVIS), showing the clearance of an intradermally injected near-infrared tracer (P20D800) that is exclusively drained by lymphatic vessels in K14-VEGF-A transgenic mice, previously treated with either F8-SIP or F8-VEGF-C, on day 74 after challenge. (**b**) Clearance rate and (**c**) half-life analysis of the P20D800 tracer in F8-SIP and F8-VEGF-C-treated mice 74 days post-challenge (expressed as mean clearance rate resp. half-life, *n* = 4 mice per group, two-tailed Student’s *t*-test). (**d**,**e**) Representative immunofluorescence images of ear sections from F8-SIP and F8-VEGF-C-treated mice stained for CD45 (red, (**d**)) and quantification of the CD45^+^ area in the region of interest of F8-SIP and F8-VEGF-C-treated mice after resolution of inflammation ((**e**), expressed as mean %, *n* = 8–9 mice per group, two-tailed Student’s *t*-test). (**f**,**g**) Representative immunofluorescence images of CD68 (green, (**f**)) and quantification of the CD68^+^ area in the region of interest (**g**) of F8-SIP and F8-VEGF-C-treated mice after resolution of inflammation (expressed as mean %, *n* = 8–9 mice per group, two-tailed Student’s *t*-test). Dotted yellow line: basement membrane (BM). Size bars: 50 μm. (**h**–**j**) Flow cytometry-based quantification of (**h**) CD45, (**i**) CD4, and (**j**) CD8-positive cells in F8-SIP and F8-VEGF-C-treated VEGF-A transgenic mice 74 days post-challenge (expressed as % of all living cells, *n* = 4 mice per group, two-tailed Student’s *t*-test). All data are presented as mean ± SD.

Next, we examined the immunological landscape and lymphatic drainage functionality of F8-SIP and F8-VEGF-C-treated K14-VEGF-A transgenic ear skin after the inflammation had resolved. First, we assessed the drainage capacity of the lymphatic vasculature in the ears in both groups by tracking the clearance of an intradermally injected, lymphatic-specific near-infrared tracer (P20D800) [17]. We determined the clearance rate and half-life of the injected tracer over the course of 6 h at 74 days post-challenge but found no significant difference (Figure 2a–c). By immunofluorescence, we also analyzed the distribution of immune cells in the ear skin. No significant changes in the density of CD45^+^ cells in F8-SIP or F8-VEGF-C-treated K14-VEGF-A transgenic mice were detected after 74 days (Figure 2d,e), and there were no significant changes in macrophage distribution, as defined by CD68 (Figure 2f,g), CD206, and F4/80 (Supplementary Figure S2a–c). Congruently, using flow cytometry of whole ears at day 74 in both treatment groups, we found no significant differences in CD45^+^ immune cell infiltration (Figure 2h, Supplementary Figure S2d) and CD4^+^ or CD8^+^ T cell numbers between ears of both treatment groups (Figure 2i,j, Supplementary Figure S2e), suggesting that the immunological status of the ear skin was comparable in both groups at this timepoint. 

### 3.3. Prior Treatment with the F8-VEGF-C Fusion Protein Results in Reduction of Ear Skin Edema upon Re-Challenge

**Figure 3:**
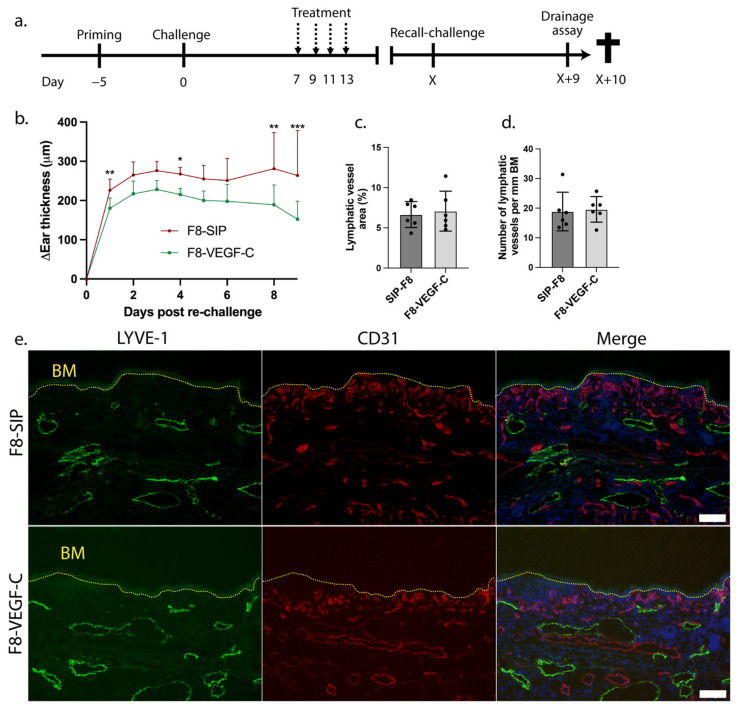
(**a**) Schematic representation of the long-term inflammation experimental setup with the treatment regimen on days 7, 9, 11, and 13 (dashed arrows). X indicates the day when the ear thickness of both treatment groups had returned to baseline, and therefore the day when inflammation was resolved. (**b**) Changes in ear thickness over 10 days after re-challenge in F8-SIP and F8-VEGF-C-treated K14-VEGF-A transgenic mice relative to ear thickness before re-challenge (*n* = 4–5 mice per group, 2-way ANOVA with Bonferroni post-test; one representative of two independent experiments shown). (**c**) Quantitative analysis of lymphatic vessel area as % of the region of interest at day 10 after re-challenge (expressed as a mean %, *n* = 6 mice per group, two-tailed Student’s *t*-test, one representative of two independent experiments shown). (**d**) Analysis of number of lymphatic vessels in previously F8-SIP and F8-VEGF-C-treated K14-VEGF-A transgenic mice on day 10 after re-challenge (expressed as mean number per mm basement membrane length, *n* = 6 mice per group, two-tailed Student’s *t*-test, one representative of two independent experiments shown). (**e**) Representative immunofluorescence images of ears from F8-SIP and F8-VEGF-C-treated K14-VEGF-A transgenic mice stained for Lyve-1 (green), CD31 (red), and Hoechst 33342 (blue) on day 10 after re-challenge. Dotted yellow line: basement membrane (BM). Size bars: 100 μm. All data are presented as mean ± SD. * *p* < 0.05, ** *p* < 0.01, *** *p* < 0.001.

**Figure 4:**
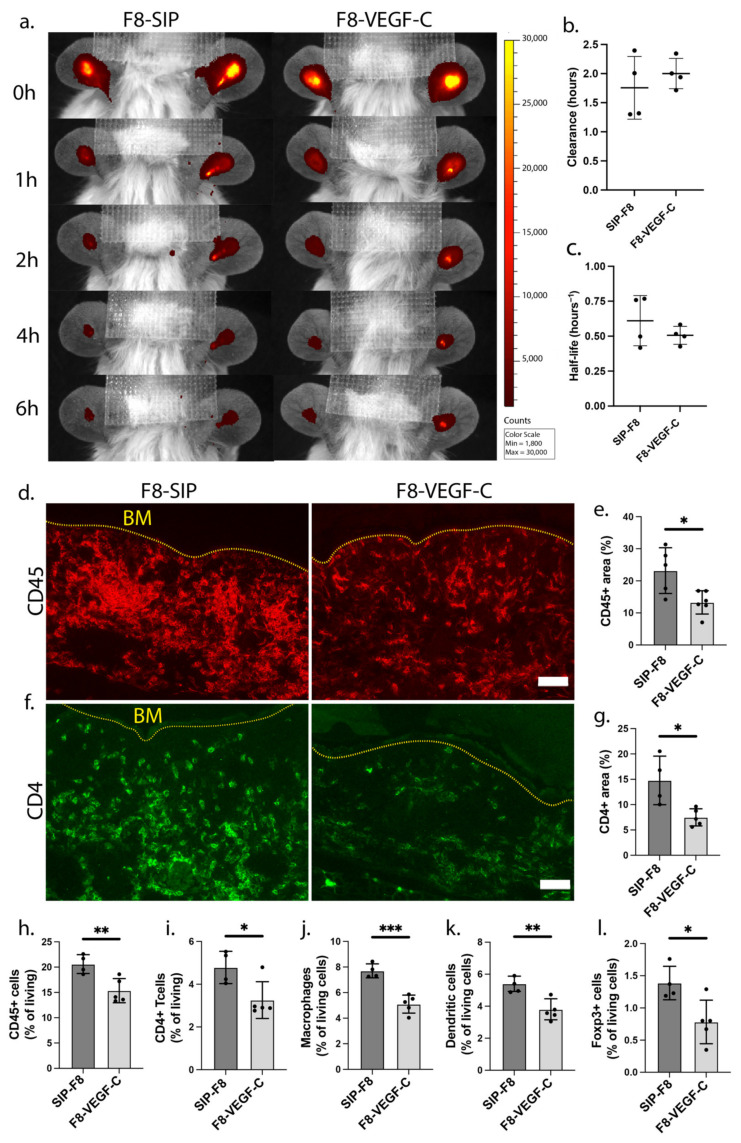
(**a**) Representative IVIS-images showing clearance of the intradermally injected near-infrared dye P20D800 in K14-VEGF-A transgenic mice initially treated with either F8-SIP or F8-VEGF-C, 10 days after re-challenge. (**b**) Clearance rate and (**c**) half-life of the P20D800 tracer in F8-SIP and F8-VEGF-C-treated K14-VEGF-A transgenic mice 10 days after re-challenge (expressed as mean clearance rate resp. half-life, *n* = 4 mice per group, two-tailed Student’s *t*-test). (**d**,**e**) Representative immunofluorescence images (**d**) and quantification (**e**) of ears from F8-SIP and F8-VEGF-C-treated K14-VEGF-A transgenic mice stained for CD45 (red) (expressed as mean %, *n* = 5–6 mice per group, two-tailed student’s *t*-test, one representative of two independent experiments is shown). (**f**,**g**) Representative immunofluorescence images (**f**) and quantification (**g**) of ears stained for CD4 (green) (expressed as mean %, *n* = 5–6 mice per group, two-tailed Student’s *t*-test, one representative of two independent experiments is shown). (**h**–**l**) Quantification of total CD45^+^ cells (**h**) and immune cell subsets (**i**) CD4^+^ Foxp3^-^ (T helper cells), (**j**) CD11b^+^ F4/80^+^ (macrophages), (**k**) CD11c^+^ MHCII^+^ (dendritic cells), and (**l**) CD4+ Foxp3^+^ (regulatory T cells) in F8-SIP and F8-VEGF-C-treated K14-VEGF-A transgenic mice 10 days after re-challenge (expressed as % of all living cells, *n* = 4–5 mice per group, two-tailed Student’s *t*-test, one representative of two independent experiments shown). Dotted yellow line: basement membrane (BM). Size bars: 50 μm. All data are presented as mean ± SD. * *p* < 0.05, ** *p* < 0.01, *** *p* < 0.001.

To investigate a potential long-term protective effect of the F8-VEGF-C fusion protein treatment, we induced secondary inflammation in K14-VEGF-A transgenic mice after primary inflammation had resolved. To this end, we applied the same hapten and monitored the ear thickness for 10 days post-re-challenge (Figure 3a). Importantly, mice that had previously been treated with F8-VEGF-C showed a significant reduction of ear thickness compared to their F8-SIP-treated counterparts, even though the mice had been treated more than 60 days before. This effect was maintained during the whole observation period (Figure 3b), demonstrating the long-term protective activity of prior VEGF-C treatment in recurrent inflammation. By immunofluorescence, we then aimed to investigate changes in lymphatic vessel density between the two treatment groups. Staining ear sections of F8-SIP and F8-VEGF-C-treated K14-VEGF-A transgenic mice for Lyve-1, we found no major differences in lymphatic vessel density in the inflamed ear skin between both treatment groups at day 10 after re-challenge (Figure 3c–e). To elucidate whether the reduction in ear thickness of K14-VEGF-A transgenic mice previously treated with F8-VEGF-C upon re-challenge resulted from the persistently enhanced lymphatic network and therefore from an increased lymphatic drainage capacity, we again determined the clearance rate and half-life of the P20D800 lymphatic tracer over the course of 6 h (Figure 4a). There was no significant difference between the groups on day 10 post-re-challenge (Figure 4b,c).

### 3.4. Treatment with the F8-VEGF-C Fusion Protein Results in Significant Reduction of Immune Cell Infiltration upon Inflammatory Re-Challenge

Finally, we investigated the influence of prior F8-VEGF-C treatment on immune cell distribution in re-challenged ears of K14-VEGF-A transgenic mice. Strikingly, tissue sections stained for CD45 revealed a significant reduction in the CD45^+^ area in the skin of F8-VEGF-C-treated mice (Figure 4d,e). In addition, prior F8-VEGF-C treatment also reduced the CD4^+^ area (Figure 4f,g), suggesting reduced T cell infiltration. To further confirm and expand those findings, we investigated immune cell infiltration in re-challenged ears by flow cytometry. In agreement with the immunofluorescence results, CD45^+^ and CD4^+^ cells were significantly reduced in previously F8-VEGF-C-treated mice (Figure 4h,i, Supplementary Figure S3a,b). Additionally, F8-VEGF-C treatment resulted in a significant decrease in the infiltration of a variety of further immune cell populations, including macrophages (CD11b^+^, F4/80^+^) and dendritic cells (CD11c^+^, MHCII^+^) (Figure 4j,k, Supplementary Figure S3c,d). Interestingly, regulatory T cells, identified as CD4^+^ Foxp3^+^ cells, were also significantly decreased 10 days post-rechallenge (Figure 4l, Supplementary Figure S3e). All in all, these data demonstrate that treatment with the F8-VEGF-C had a long-term anti-inflammatory effect and reduced both edema and immune cell infiltration upon recurrent inflammation at the same site.

## 4. Discussion

Although several agents to treat chronic inflammatory diseases such as psoriasis exist, most treatments are symptomatic only. Treating recurrent inflammatory episodes, however, has remained a challenge. In this study, we have demonstrated the long-term therapeutic potential of targeted VEGF-C delivery to the site of skin inflammation in a psoriasis-like dermatitis model in mice. Of note, we could show that the application of the F8-VGEF-C fusion protein not only reduced acute inflammation but also had a long-term anti-inflammatory effect against inflammatory re-challenge at the same site. This finding indicates that local induction of lymphangiogenesis could be a new approach for a potentially curative (or at least long-term effective) treatment of recurrent inflammatory diseases.

We used the contact sensitizer oxazolone to elicit a CHS response in hemizygous VEGF-A overexpressing mice under the control of the keratin-14 promoter, resulting in chronic dermatitis with ear skin edema persisting over 2 months. This model faithfully reproduces several psoriasiform symptoms, such as skin reddening, epidermal hyperplasia, tortuous vessels, and immune cell infiltration [14,19,20,21,22]. After consecutive treatments with the F8-VEGF-C fusion protein, we observed a significant reduction in ear skin edema in inflamed K14-VEGF-A transgenic mice. These findings are in line with our previous data with this fusion protein [6] and with studies where local stimulation of the VEGF-C/VEGFR-3 axis attenuated chronic inflammatory conditions [9,12,23].

The most striking observation we made was the long-lasting effect of targeted VEGF-C delivery to the site of skin inflammation. The expanded dermal lymphatic system in F8-VEGF-C-treated mice persisted for more than 170 days. Similar effects have been obtained by transgenic induction of VEGF-C in the skin of adult mice for 1 to 2 weeks, which resulted in persistent lymphatic expansion that lasted over 6 months [24], suggesting that the lymphatic network, once expanded, is fairly stable, at least in the skin. This persistent lymphatic expansion in F8-VEGF-C-treated mice resulted in significantly reduced ear skin edema and immune cell infiltration upon inflammatory re-challenge. Consequently, we believe that the long-term anti-inflammatory effects of the F8-VEGF-C fusion protein might represent a novel therapeutic approach for chronic inflammatory diseases such as psoriasis, rheumatoid arthritis, or inflammatory bowel disease. These conditions are well known to recur/relapse frequently [25,26,27]. Especially in the case of psoriatic skin lesions, recurrence often occurs at the same anatomical location [25]. Thus, treatment with the fusion protein could potentially prevent or ameliorate future inflammatory episodes. Furthermore, compared to TNF-alpha blockade, the F8-VEGF-C fusion protein showed equal efficacy in chronic inflammatory mouse models [6]. Other therapeutics for psoriasis include topical formulations such as corticosteroids, vitamin D3 analogues, calcineurin inhibitors, and keratolytics [28,29]. However, incomplete treatment adherence has been shown to limit the effectiveness of these therapeutics [30]. Thus, local VEGF-C treatment appears as an attractive new approach with a low side effect profile and additional long-term anti-inflammatory activity that may protect from recurrence. Further, recombinant proteins in clinical setups are mostly applied in a professional setup, ensuring patient adherence.

To identify the underlying mechanism of reduced ear skin edema and immune cell infiltration, we performed a lymphatic clearance assay, as well as immunofluorescence and flow cytometry analyses of tissue samples. Previous studies have demonstrated that treating acute inflammation with VEGF-C increases local drainage of interstitial fluid, inflammatory mediators, and inflammatory immune cells [6,8]. Here, we could not detect an increased drainage capacity despite an increased lymphatic network in the previously F8-VEGF-C-treated mice compared to the F8-SIP control mice both before and after inflammatory re-challenge. Potentially, the short half-life of the tracer we used (P20D800) did not allow us to detect small changes in drainage capacity. Therefore, additional studies using a tracer that is cleared more slowly from the affected tissue are warranted.

An additional mechanism could be that F8-VEGF-C alters the skin microenvironment via the enhanced lymphatic network. It has been reported that VEGF-C-mediated lymphatic expansion in the skin leads to immune modulation and inhibits the maturation of dendritic cells upon inflammation in vitro [31]. Additionally, LECs produce TGF-β1 and prostacyclin, two soluble immune-inhibitory molecules [32]. These factors might contribute to the long-lasting, anti-inflammatory effects of F8-VEGF-C treatment. In addition, skin-resident LECs up-regulated programmed cell death-1 ligand 1 (PD-L1) upon oxazolone-induced CHS [33], which may result in the inhibition of T cells within the skin or recirculating towards draining LNs [34]. 

The D6 chemokine receptor is expressed by LECs and mediates the reduction of immune cell infiltration into tissues by scavenging inflammatory chemokines. Studies have shown that knocking out the D6 receptor in mice leads to an increased recruitment of immune cells [35]. An enhanced lymphatic network like in the F8-VEGF-C-treated mice could therefore reduce inflammation also by chemokine scavenging. 

Inflammation-induced junctional remodeling in initial lymph vessels has been shown in models of pneumonia [36] and dermal vaccinia virus infection [37]. Importantly, zippering of endothelial junctions has been suggested to decrease the permeability of initial lymphatic vessels. Similarly, VEGF-C treatment has been shown to induce zippering of lacteals [38]. Thus, the F8-VEGF-C fusion protein could potentially influence junctional remodeling and lymphatic permeability. However, it is currently unknown if junctional remodeling is reversible, and whether zippering of lymphatic endothelial junctions would elicit a positive or negative effect on ear edema and immune cell infiltration in our model.

Finally, we have previously demonstrated that acute activation of VEGFR-3 in LECs results in downregulation of several immune-response related genes, including adhesion molecules, chemokines, and interferon-regulated genes [39]. This means that F8-VEGF-C itself might have a broad, lymphatic expansion-independent anti-inflammatory effect on LECs. However, these VEGF-C-mediated transcriptional changes towards an anti-inflammatory profile would have to persist in LECs in order to explain the reduced severity of inflammation upon re-challenge.

Collectively, we have shown a long-lasting, anti-inflammatory effect of the F8-VEGF-C fusion protein in a model of recurrent skin inflammation. While the exact mechanisms of action have only been partially revealed and further studies will need to be performed, we believe that the persistently enhanced lymphatic density in the VEGF-C-treated group dampens the initial inflammation upon secondary hapten challenge, explaining the difference in ear thickness and immune cell infiltration. Thus, therapeutic induction of persisting LVs may be a strategy to prevent future inflammation at the same anatomical site.

## 5. Patents

US2021163579 (A1)—F8-VEGF-C.

## Data Availability

Not applicable.

## Supplementary Materials

The following supporting information can be downloaded at: https://www.mdpi.com/article/10.3390/cells12010172/s1, Figure S1: Dermal lymphatic vessels on day 179; Figure S2: Macrophage analysis and gating strategy long-term; Figure S3: Gating strategy re-challenge.
