# Supplementary material for: Antibody-Mediated Delivery of VEGF-C Promotes Long-Lasting Lymphatic Expansion That Reduces Recurrent Inflammation
Source: Cells. 2022 Dec 31;12(1):172. doi: 10.3390/cells12010172 (PMC9818868; doi:10.3390/cells12010172)
Supplement: Supplementary file 1 [file cells-12-00172-s001.zip › Supplementary Figure S1.pdf]

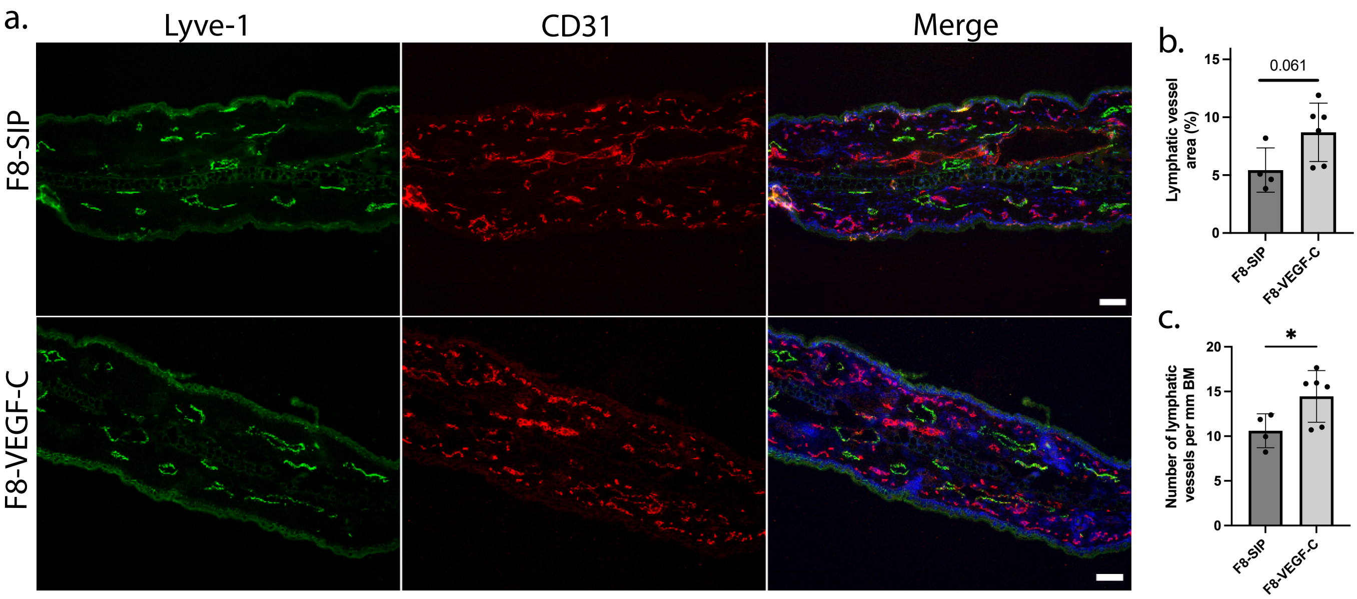

Supplementary Figure S1. (a) Representative immunofluorescence images of ears from F8-SIP and F8-VEGF-C treated mice stained for the lymphatic marker Lyve1 (green), the vessel marker CD31 (red) and Hoechst 33342 (blue) on day 179 post-challenge. Size bars: 100µm (b) Quantification of lymphatic vessel area in percentage of the analyzed region of interest in F8-SIP and F8-VEGF-C treated mice on day 179 post-challenge (expressed as mean percentage, n=5 mice per group, 2 tailed student's t-test). (c) Analysis of the number of lymphatic vessels in F8-SIP and F8-VEGF-C treated mice in the region of interest on day 179post-challenge (expressed as mean number per mm basement membrane length, n=5 mice per group, 2 tailed student's t-test).
All data are presented as mean ± SD. *P < 0.05
