# Supplementary material for: Antibody-Mediated Delivery of VEGF-C Promotes Long-Lasting Lymphatic Expansion That Reduces Recurrent Inflammation
Source: Cells. 2022 Dec 31;12(1):172. doi: 10.3390/cells12010172 (PMC9818868; doi:10.3390/cells12010172)
Supplement: Supplementary file 1 [file cells-12-00172-s001.zip › Supplementary Figure S2.pdf]

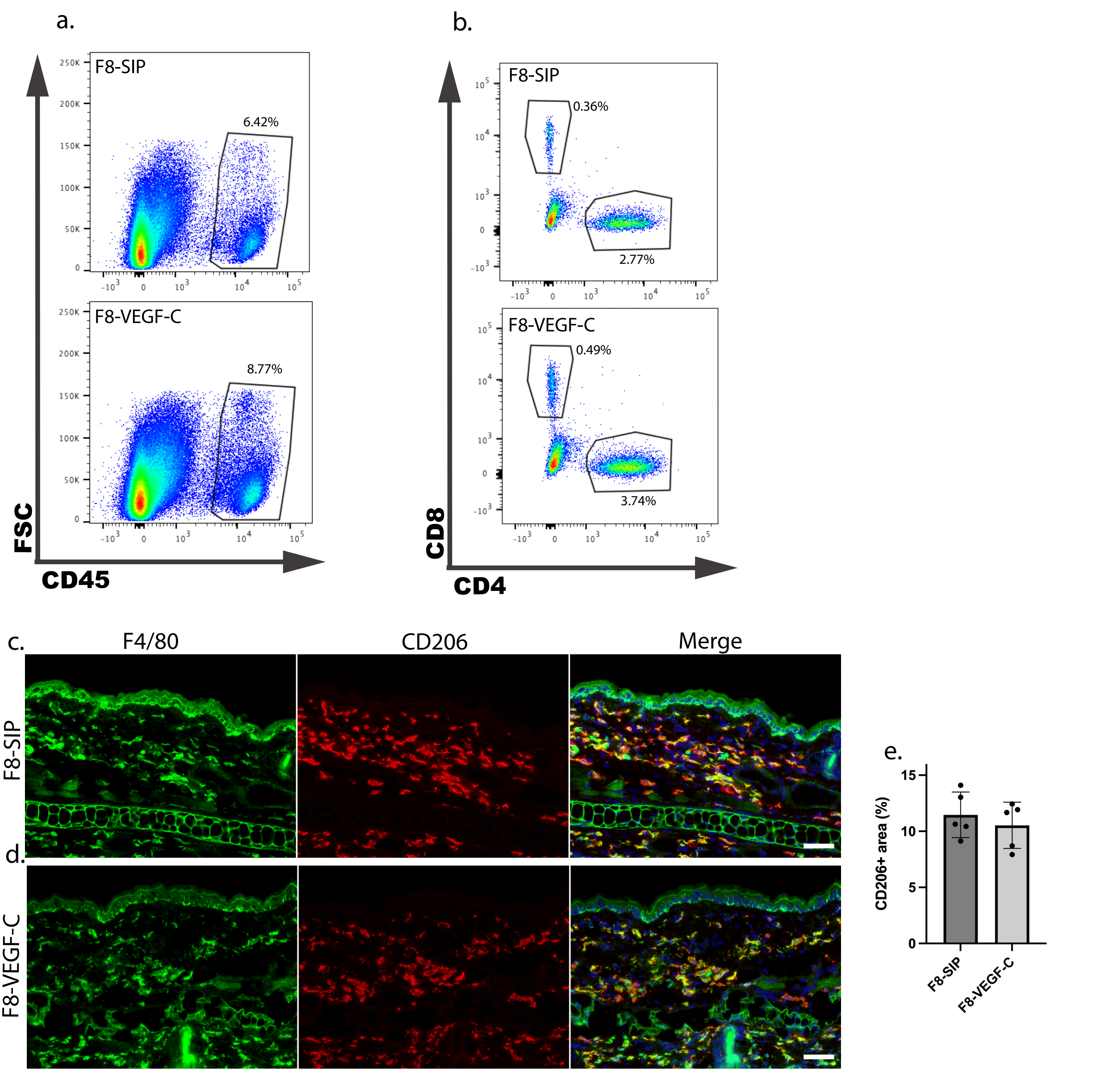

Supplementary Figure S2. Representative FACS plots with gating strategy. Data is presented as % of all living single cells of ears in either F8-SIP or F8-VEGF-C treated mice. Gating strategy for (a) CD45+ and (b) CD4+ resp. CD8+ Tcells. (c,d) Representative immunofluorescence images of ear sections from F8-SIP and F8-VEGF-C treated mice stained for F4/80 (green), CD206 (red) and Hoechst (blue) (e) and quantification of the CD206+ area in the region of interest of F8-SIP and F8-VEGF-C treated mice after resolution of inflammation expressed as mean percentage, n=5 mice per group, 2 tailed student's t-test).
