# Supplementary material for: Antibody-Mediated Delivery of VEGF-C Promotes Long-Lasting Lymphatic Expansion That Reduces Recurrent Inflammation
Source: Cells. 2022 Dec 31;12(1):172. doi: 10.3390/cells12010172 (PMC9818868; doi:10.3390/cells12010172)
Supplement: Supplementary file 1 [file cells-12-00172-s001.zip › Supplementary Figure S3.pdf]

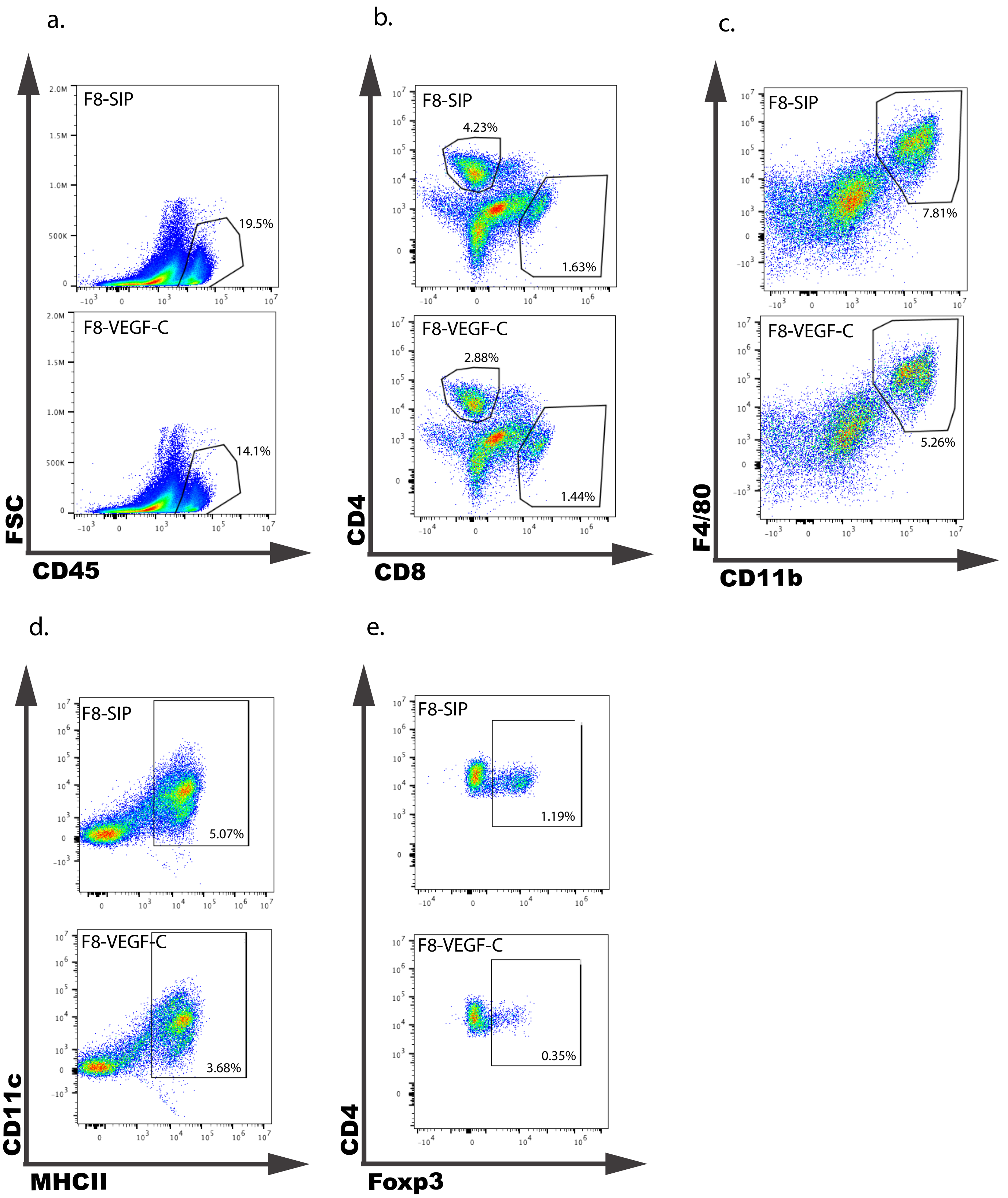

Supplementary Figure S3. Representative FACS plots with gating strategy. Data is presented as % of all living single cells of ears in either F8-SIP or F8-VEGF-C treated mice. Gating strategy for (a) CD45+ and (b) CD4+ resp. CD8+ T cells. Representation of (c) F4/80+/CD11b+ macrophages, (d) CD11c+/MHCII+ dendritic cells and (e) Foxp3+/CD4+ regulatory T cells.
